# Excesses of Modern Times and Their Relation to Disease

**Published:** 1908-04

**Authors:** Marion McH. Hull

**Affiliations:** Atlanta, Ga.


### EXCESSES OF MODERN TIMES AND THEIR RELATION TO DISEASE.*

BY MARION MCH. HULL, M. D., ATLANTA, GA.

One of the most prevalent causes of impaired health in this latter day is the indulgence in one or more excesses. We frequently have various forms of disease resulting from this cause whose symptoms are so complex that to describe them in detail would almost exhaust the list of known symptoms. For instance, there occurs to my mind as being very prevalent conditions nephritis, paresis, and gouty or rheumatoid conditions. It is not our purpose now to present an exhaustive study of how these originated, but to suggest in one or more instances the underlying causes and their development, hoping that it will help us to avoid such conditions in the future, by removing the possible cause now.

Underlying all diseases are four essential causes, as follows:

---

*Read before the Fulton County Medical Society, Feb. 20, '08

Inherited diatheses, nutritive disorders, nerve reaction, and infections. Rarely does one of these occur alone, but more often a combination of two or more becomes operative, and disease is the result. For example, take a child born of tuberculous parents; it is entirely possible for that child to develop into the most vigorous manhood and never have a sign of tuberculosis, even though it has an inherited diathesis. Suppose, however, that about the age of puberty when the whole nervous system is under great strain to control the functions properly, when the whole nature is undergoing a revolution, the child should eat some food which is difficult of digestion. a nutritive disorder would ensue and the resistance of the child be lowered. Now suppose this child at the time is in contact with tubercular bacilli exhaled from the lungs of a tuberculous parent. During all these years he has been inhaling this infected atmosphere, but has not developed the disease; now, because of the nerve reaction above spoken of, the disordered nutrition, with consequent lowered resistance, the bacilli gain foot-hold in a favorable soil furnished by the inherited diathesis, and the disease results. Thus we see that all four of these essential causes frequently enter into the etiology of a particular disease.

Of these four causes, however, what most concerns us in our present study is the influence exerted by nutritive disorders and nerve reactions. I propose to limit our discussion to two particular diseases which are very prevalent, and of which, I feel convinced, excesses are very prevalent causes; I refer particularly to arterio-sclerosis, with its multiform manifestations, and gouty affections. Take for instance, arterio-sclerosis; it consists essentially of a degenerative condition of the arteries of the body, the elastic fibres at first undergoing a fatty degeneration, weakening the walls and subsequently a sclerotic or hardened condition taking its place, the normal resilient tissues being replaced by non-elastic tissues. Now various symptoms may result from this, depending upon the degree of sclerosis and the location of it. Should the arteries of the kidneys be affected, the symptoms of nephritis would result. An improper elimination of the products of metabolism which should be excreted by the kidneys would bring on a train of symptoms, which might produce subsequent heart or mental disorders. Should the arteries of the brain be affected, apoplexy would result, with immediate death, or hemiplegia with local softening of the brain; or paresis, might result with all its attendant train of symptoms. If the arteries of the heart should become affected, severe attacks of angina pectoris might follow. So you see that underlying a great many diseases is this condition of arterio-sclerosis.

Most authors in writing on this subject, assign as the principal cause alcoholism or syphilis. Of course, these diseases may, and undoubtedly do, produce arterio-sclerosis; but it has been my experience to treat a great many cases in which neither of these causes could be assigned; in fact, 90 per cent. could not be traced to either of them. In trying to account for the conditions, I have come to the conclusion that excesses of eating and of working, have been more responsible for them than the worship of Bacchus and Venus. Take for instance, the matter of over-eating; we all over-eat; it requires an amount of food which will furnish three thousand calories to supply the demands of the body for twenty-four hours, and this should be in the proportion of about 100 grms. of proteid, 600 grms. of carbo-hydrates, and 60 grms. of fats. This would be furnished by about four ounces of meat, or three glasses of milk and two eggs; about six to eight thin slices of bread, an ordinary serving of potatoes and rice, a dish of cereal morning and evening; an ordinary portion of butter at each meal; and from six to eight glasses of fluid in the twenty-four hours. A cursory glance at this will show in a moment that we ordinarily eat too large a proportion of meat, drink too little fluid, and eat too little fruit; and on the whole eat a great deal more than is required. The effect of eating too much would be to require the system to do more work and the eliminative organs to overtax themselves in their attempt to get rid of the products of metabolism, which should be excreted. Where exercise is deficient the organs are not able to eliminate these products, and a general accumulation takes place. These poisonous products circulating in the blood have the same effect on the cells of the body as the poisons of alcohol or of syphilis. The cells forming the arterial walls are fed by food laden with poisonous products; degeneration takes place, followed by sclerosis. In this way, excess in eating may cause arterio-sclerosis, which may manifest itself in nephritis, apoplexy, or paresis. This is not mere theory. I recall now, a case of a gentleman who was most temperate in every respect except in the matter of eating; he was an enormous eater, and died of apoplexy after a few hours illness. There was no other way to account for the sclerosis in this case, except as above. I have also in mind a number of cases of nephritis which cannot be traced to any other origin. It is a matter of common knowledge that excesses in eating cause gouty conditions; so frequently is gout associated with the rich and overfed, that we have commonly supposed that it could not occur in the poor and underfed; which, however, is an error. In these latter, however, a different series of phenomena account for the error of metabolism.

Another one of the excesses which is extremely common and which is a very prevalent source of disease, is mental excess. By this I do not mean an excess of mental work only, but worry associated with work. Work wearies, but worry wears out. The fierce competition of modern times and the over-weaning desire to augment wealth has developed a condition of high tension, that eventually results in disease of this character. Let us see how this comes about; a man now-a-days gets up, dresses hurriedly, eats his breakfast in haste, not chewing his food properly and frequently at the same time planning his day's work, withdrawing the blood to the brain from the digestive organs where it is needed for proper functionating; he hurries forth immediately after eating, and plunges head-long into a rush of work. At lunch, tired and with deficient nerve power, he hurries home, and through another meal in the same fashion, and back to his desk. Then when evening comes he is very much exhausted, and not able to properly digest the food which he ingests; and going to bed with his business on his mind, is fortunate if he is able to rest quietly. The next day the same round is repeated. If he is of that happy disposition that enables him to accomplish his work without worry, he is able to stand this exhaustive demand for quite awhile; but there are very few men who have that gift, and the little worries wear out the already over-taxed nervous system. Then the element of nerve reaction comes into play, and with insufficient nerve power, nutritive disturbances result, with increased formation of the products of metabolism, and decreased elimination, with consequent defective nourishment of the cells, with degenerative conditions, and sclerosis. This is not purely theoretical either; for I have in mind at the present time several cases of this condition.

When we have advanced thus far, we have shown that the excesses in eating and in mental work with worry, are capable of producing disease, and we have shown how and why they do. It is not necessary to demonstrate that the more common and vulgar forms of excess would develop disease conditions; so that we are prepared to take the next step—how it may be prevented. I would suggest, first, that we can be more frugal in our meals than we have been in the past. I was recently at an institution where I had the pleasure of being thrown with a company of as fine a lot of men physically and otherwise as it has been my pleasure to see in many a day. Their breakfast consisted of a cup of coffee and of a bowl of oat meal, bread and butter, and boiled mutton; their dinner of chicken, baked potatoes, bread and gravy, and stewed fruit; and their supper of crackers and milk. Comparing this meal with what we ordinarily indulge in, and comparing the vigorous physique of these young men with that of the pampered rich, we can but feel that the advantage is in favor of frugality; certainly from an economical standpoint it would be so. Another thing we would learn would be that less worry and work would accomplish the same thing, if we combined with it a third suggestion, that I should like to make, that there should be more time given to outdoor exercise and recreation. I believe a revolution would be worked in the health of our community, their lives would be prolonged and life made more joyous if a specified time would be given to recreation and rest. The outdoor exercise would stimulate the excretory organs and improve the metabolism so that the various functions of the body would not be taxed beyond their powers. The nervous system would be better, and improved health would result. I furthermore believe, that with the general health improved, a larger amount of business could be accomplished in a shorter number of hours; and instead of financial loss resulting from the time given to recreation, financial gain would be the result. This lesson is particularly necessary for us in the South, at this time, with its marvellous material development. We have attempted to keep up with its progress, but have not yet learned the secret of working intensively for a short time and stopping. To illustrate; in Atlanta, the average business man is in his office at seven-thirty and works until six-thirty or seven in the evening at least, crowding in every hour with its full quota of work; in the Northern cities, scarcely any one is in his office until nine o'clock and by four or five the offices are vacated. He works as intensively while he is at the office as does the alert Atlanta man, but he does not work as long; he has learned better how to take care of himself. I think enough has been said now to present my case; my plea is for a readjustment of our life and recognition of what the body requires, what its capabilities and possibilities are, and that we give the individual self a square deal, not expecting more work than God required of it to perform. I believe that the present rate of living is developing an increased number of cases of nephritis, arterio-sclerosis and rheumatoid conditions; it is time that we called a halt.

